# EUS-Guided Vascular Procedures: A Literature Review

**DOI:** 10.1155/2013/865945

**Published:** 2013-05-07

**Authors:** Tomislav Bokun, Ivica Grgurevic, Milan Kujundzic, Marko Banic

**Affiliations:** ^1^Department of Gastroenterology, University Hospital Dubrava, Zagreb University School of Medicine, Avenue Gojka Suska 6, 10 040 Zagreb, Croatia; ^2^Rijeka University School of Medicine, Brace Branchetta 20, 51 000 Rijeka, Croatia

## Abstract

Endoscopic ultrasound (EUS) is continuously stepping into the therapeutic arena, simultaneously evolving in different directions, such as the management of pancreatic and biliary diseases, celiac neurolysis, delivering local intratumoral therapy, and EUS-guided endosurgery. EUS-guided vascular procedures are also challenging, considering the variety of vascular pathology, proximity of the vascular structures to the GI tract wall, high resolution, and real-time guidance offering an attractive access route and precise delivery of the intervention. The literature on vascular therapeutic EUS demonstrates techniques for the management of upper GI variceal and nonvariceal bleeding, pseudoaneurysms, and coiling and embolization procedures, as well as the creation of intrahepatic portosystemic shunts. The paucity of studies, diversity of study designs, and the number of animal model studies hamper a systematic approach to the conclusion and decision making important to clinicians and healthcare policy makers. Nevertheless, theoretical benefits and findings up to date concerning technical feasibility, efficacy, and safety of the procedures drive further research and development in this rather young therapeutic arena.

## 1. Introduction

Since its beginning in the 1980s, EUS has evolved into a powerful diagnostic tool used widely for a number of GI conditions, successfully replacing other “gold standard” diagnostic modalities. In recent years, EUS is expanding to the interventional arena, usurping the management of pathology that was traditionally in the domain of therapeutic endoscopy, interventional radiology, and surgery. Therapeutic endoscopic ultrasonography (T-EUS) is developing simultaneously in different areas, such as the management of pancreatic and biliary diseases (e.g., pseudocyst drainage, cholangiopancreatography, etc.), celiac neurolysis, delivering local intratumoral therapy, EUS-guided endosurgery, and vascular procedures [1–4]. The variety of vascular pathology and the proximity of the vascular structures to the GI tract walls call for EUS-guided vascular procedures. Additionally, high resolution and real-time guidance offer precise delivery of the intervention, which is especially important when targeting tiny vascular structures. The literature on vascular therapeutic EUS offers a wide range of procedures that will be summarized in this review. The intention of this review is to give the reader a quick overview of the current state of research of EUS-guided vascular interventions. For more detailed descriptions of various techniques in this arena, we refer the readers to the original papers cited here.

## 2. Methods

PubMed/MEDLINE was searched to identify relevant publications in English. The following search string was used: {(EUS-guided or endosonography guided or endosonographic guidance or endoscopic ultrasound guidance or endoscopic ultrasound guided or echo-endosonography guided or echo-endosonographic guidance or endoscopic Doppler US guided or endoscopic Doppler ultrasound guided or endoscopic Doppler US guidance) AND (vascular or EUS-guided or varices or nonvariceal or pseudoaneurysm or Dieulafoy or Dieulafoy's or porto-systemic shunt)}. The final search was launched on January 29, 2013, with no time restrictions. Additionally, the Cochrane Library was searched. 

Our PubMed/MEDLINE search yielded 1291 publications that were assessed for relevance according to title and abstract by two reviewers, and potentially relevant papers were retrieved in full text. The bibliographies of publications identified as relevant were manually searched for potentially relevant titles, and additional publications were found. Finally, twenty published papers were included for analysis in this review. Abstracts presented at congresses were not included. We are aware that our search possibly missed relevant papers due to a relatively inconsistent and evolving terminology in this new therapeutic arena.

In this paper, the current literature on EUS-guided vascular procedures is summarized in a narrative form considering the paucity of the studies, great diversity of study designs (mostly pilot studies, case records, and small case series), and a number of animal model studies, hampering any systematic approach to the literature synthesis.

We were restricted to the literature on vascular procedures under real-time EUS guidance. There are various miniature ultrasound/Doppler probes available that can be introduced through a working channel of the standard endoscope [5, 6]. Studies describing the treatment with the aid of endoscopic Doppler ultrasound with Doppler probes or ultrasound miniature probes, which did not occur under real-time EUS guidance, are worth mentioning but were not included in this review [6–12]. Studies describing non-GI tract-related EUS-guided procedures were also not included.

## 3. Results and Discussion

Several areas of vascular procedures under the EUS-guidance could be identified throughout the available literature. For the purpose of this review, we have selected five areas of EUS-guided vascular procedures:

1. EUS-guided management of nonvariceal upper GI bleeding;
2. EUS-guided management of variceal bleeding;
3. EUS-guided management of pseudoaneurysms;
4. EUS-guided embolization of portal venous system;
5. EUS-guided creation of portosystemic shunts.

In the next sections, each of these areas will be discussed overviewing the available literature.

### 3.1. EUS-Guided Management of Nonvariceal Upper GI Bleeding

Since a certain percentage of nonvariceal upper GI bleeding episodes are diagnostically challenging and refractory to a standard therapy (various endoscopic techniques, angiographic therapy), EUS-guided detection and management can offer an alternative in this group of patients. To date, EUS-guided therapy for nonvariceal upper GI bleeding has been described for the management of peptic ulcer disease, Dieulafoy's lesions, and bleeding tumors. EUS-guided management of the pseudoaneurysms, which also can bleed, will be discussed in the following section of this review.

Peptic ulcer disease is responsible for more than 50% of upper GI bleeding admissions to hospitals and has high recurrent bleeding rates and high mortality rates. Standard treatment, including nonendoscopic modalities, and upper GI endoscopy with injection therapy, thermal therapy, and/or clipping are safe and effective. However, relatively high rates of unsuccessful bleeding stoppage and recurrent bleeding are reported, occurring in up to 20% of patients [13–15]. EUS guided therapy offers therapeutic techniques that can potentially improve successful treatment, especially in the group of patients with unsuccessful bleeding stoppage and recurrent hemorrhage, therefore reducing recurrent bleeding rates and high mortality rates. 

Levy et al. described a patient with recurrent upper GI bleeding from a duodenal ulcer previously treated with heater probe plus injection at two separate occasions. On EUS-examination, the authors visualized tortuous vessel branching from gastroduodenal artery to mucosa and, under the guidance of curved linear echoendoscope, they injected 3 mL of cyanoacrylate through a 22-gauge FNA needle pointing at the 1.5 mm wide branching vessel located within the ulcer. Success of the treatment was confirmed immediately by Doppler. There were no complications, and no rebleeding occurred during 14-month followup [16]. Elmunzer et al. in their animal pilot study created an artificial arterial bleeding model in the stomach (gastroepiploic vascular bundle was surgically placed in the submucosa). After endosonographic visualization of submucosal artery, dilute epinephrine injection or contact thermal coagulation were delivered directly to the vessel through the working channel of the forward-viewing echoendoscope under direct EUS guidance. The procedure was successful in both cases treated with epinephrine injection and in two out of four treated with contact thermal coagulation [17]. Gonzalez et al. successfully treated a patient who bled from the side branch of gastroduodenal artery with EUS-guided injection of cyanoacrylate, with no recurrence bleeding detected during 14-month followup [18].

Dieulafoy's lesion is a relatively rare vascular malformation that presents with an acute refractory often massive upper GI bleeding, with a rather unsatisfactory detection rate at repeated endoscopy in the case of nonactive bleeding at the time of endoscopy. Endoscopic ultrasound and/or Doppler ultrasound are highly sensitive for the detection of vascular structures in the GI tract wall, which is especially useful in the absence of an endoscopically visible lesion (e.g., ulcer or protruding vessel), giving theoretical advantages to EUS-guided detection and treatment over standard modalities. 

Fockens et al. in their small study treated three patients with Dieulafoy lesions by EUS-guided sclerotherapy [19]. Under the direct guidance of rotating sector scanner EUS aided by an endoscopic picture, a 23-gauge needle was used to inject epinephrine/polidocanol in the lesions. All the procedures were successful without complications, with rebleeding episodes in one of the patients that were followed up. In the case record by Ribeiro et al., residual artery was detected by EUS with Doppler five months after local therapy of a Dieulafoy's lesion with rubber bands. Hereafter, thermal contact therapy following injection of absolute alcohol under direct EUS guidance successfully stopped blood flow in the artery, which was confirmed by Doppler [20]. Gonzalez et al. also reported successful EUS-guided 19-gauge injection therapy of two Dieulafoy lesions with no recurrence of bleeding during median followup of nine months [18]. Levy et al. reported a patient with recurrent active upper GI bleeding from duodenum. No bleeding site was detected throughout multiple upper GI endoscopies, and random treatment with heater probe plus injection was undertaken at three occasions without success. On EUS, a 0.8 mm vessel branching from larger underlying vessel to the duodenal mucosa was visualized. Through a 22-gauge needle, 99% alcohol was delivered, and a band was placed over the site of injection. There were no complications and no rebleeding occurred during 23-month followup [16]. 

The current evaluation of EUS-guided interventions for the management of peptic ulcer bleeding and Dieulafoy's lesions is limited to anecdotal case records, one case series and one animal study, thus hampering any comparison with standard treatment options, and therefore recommendations cannot be given. However, the theoretical benefits of direct EUS and Doppler visualization of the “culprit” vessel responsible for the recurrent bleeding are evident, allowing precise treatment delivery and therefore possibly higher successful treatment rate in the groups of patients unsuccessfully treated or with recurrent bleeding. The feasibility and safety demonstrated in these publications are encouraging, hoping that future larger scale studies with appropriate study designs will possibly find some benefit, especially in some groups of the patients (unsuccessful treatment, recurrent bleeding episodes).

### 3.2. EUS-Guided Management of Upper GI Varices and Variceal Bleeding

Endoscopic injection therapy and band ligation have been widely used for the management of bleeding and nonbleeding upper GI tract varices with a high success rate and with known complication rates [21, 22]. Yet a considerable percentage of patients are not managed successfully, having recurrent variceal bleeding [22, 23]. EUS has emerged as a valuable tool for the diagnosis, treatment planning, evaluation of treatment success, and estimation of recurrent bleeding potential, being able to visualize varices, perforating, and collateral veins, thus allowing to predict varices with a high risk for (recurrent) bleeding [24–26]. In the last years, EUS is continuously emerging as a therapeutic method for the management of upper GI varices, as guidance for injection therapy and coiling, or their combination. There are reports demonstrating feasibility, efficacy, and low complication rates of injection therapy for upper GI varices with the aid of EUS/Doppler, where EUS/Doppler was mostly used to detect and visualize the location for therapy, which was thereafter delivered through a standard forward-viewing endoscope [12, 27]. 

Treatment techniques under direct EUS guidance seem to be promising. Lahoti et al. were the first to demonstrate sclerotherapy for upper GI varices under real-time EUS guidance. In a small pilot study on five patients with esophageal varices, they injected sclerosant (sodium morrhuate) through a 2.5 mm catheter injector needle under EUS guidance directly to perforating vessels until “no flow” was detected by color Doppler [28]. To achieve varices obliteration, 2.2 sessions per patient were needed. The mean followup was 15 months, and no recurrent bleeding occurred. Romero-Castro et al. in their small case series injected cyanoacrylate-lipiodol into gastric varices at the level of perforating veins, under EUS guidance [29]. All the procedures were successful, without recurrent bleeding or other complications during followup. They postulated that targeting perforating veins would produce the maximal blood-flow blockage, with the lower amounts of cyanoacrylate needed, therefore reducing the rate of potential local and systemic complications. Gonzalez et al. in three patients from their pilot study performed EUS-guided injection therapy of varices and reported 100% success rate, without recurrent bleeding or major complications [18]. The most valuable study to date was undertaken by Andrade de Paulo et al. [30]. In their randomized controlled trial, they compared endoscopic sclerotherapy and EUS-guided sclerotherapy of esophageal collateral veins. The findings that recurrent variceal bleeding after endoscopic therapy is related to collateral veins lead them to the hypothesis that obliteration of these veins would reduce the risk of recurrent bleeding. In the first arm, 24 patients were treated with endoscopic sclerotherapy (ES group) using 3–5 mL of diluted ethanolamine oleate injected through a 23-gauge injector, depending on the size of the varix. In the second arm (EUS-ES group, 24 patients), the collateral veins were punctured under EUS guidance with 19- or 21-gauge needle, and the same sclerosant was injected. The procedures in both arms were repeated at 2-week intervals until the varices were eradicated. At the end of the interventional part of the study, EUS was performed in all patients in the first arm to detect the presence of collateral veins. The study groups did not differ in the number of procedures needed for varices eradication, total volume of sclerosant used, and pain or bleeding during/after treatment. In the ES group, eight patients (33.3%) had collaterals detected after treatment, whereas in the EUS-ES group collateral veins were undetected in any of the patients (*P* = 0.004). During followup (mean 22.6 months), four patients in the ES group (all had collateral veins detected at the end of the interventional part of the study), and two patients in the EUS-ES group (none had collateral veins detected at the end of the interventional part of the study) (*P* = 0.32), had varices recurrence without recurrent bleeding in both groups. 

**Figure 1:**
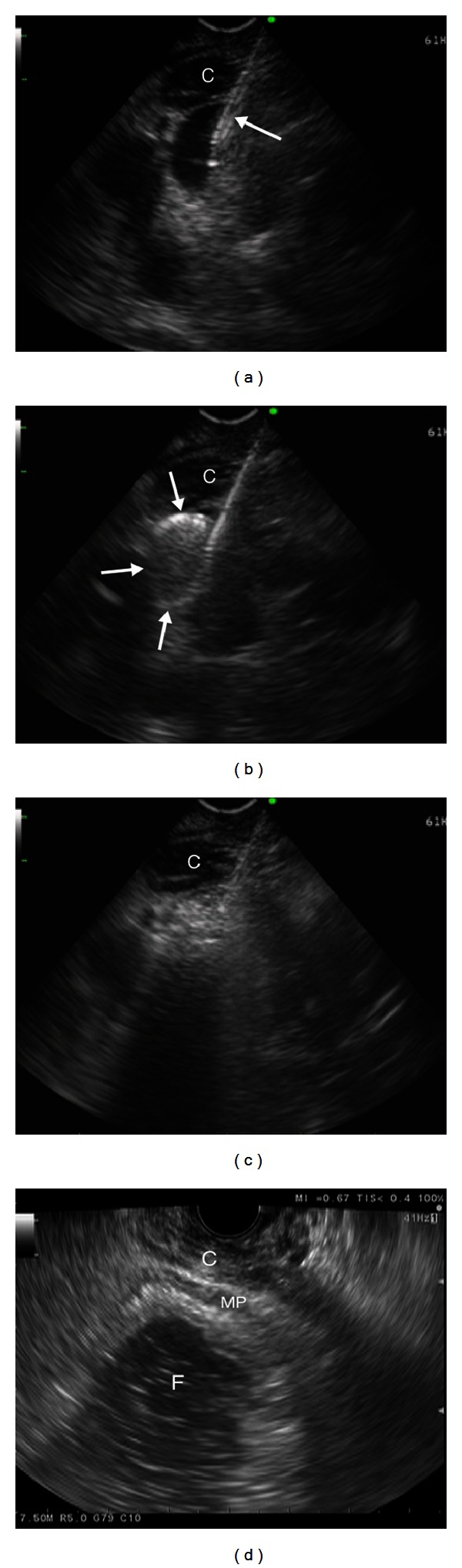
(a) Endosonographic transesophageal view—a 19-gauge needle inserted into the varix (arrow). (b) Coil delivery (arrows) through the 19-gauge needle. (c) Injection of 1 mL of cyanoacrylate. (d) Fundal varices obliterated. C: crus muscle, F: fundus; MP: muscularis propria of stomach wall. (Images curtsey of [32]).

Injection sclerotherapy for upper GI varices has been associated with local complication and embolic incidents [2, 31]. The incidence of the latter can potentially be minimized by using coils that can be introduced under EUS guidance [32]. In their case record, Levy et al. described successful EUS-guided delivery of microcoils over 22-gauge needle to three varices for the treatment of acute bleeding from ectopic coledochojejunal anastomotic varices that occurred during ERCP [33]. Romero-Castro et al. reported a small case series of patients with severe gastric varices treated with EUS-guided coil embolization. They inserted coils into the perforating veins in order to block the blood flow. The varices were eradicated in three out of four patients, and no complications occurred in the successfully treated patients during five months of followup [34]. Binmoeller et al. in their study on thirty patients combined EUS-guided coiling and cyanoacrylate glue injection by transesophageal approach to the gastric fundal varices. They hypothesized that coils with attached synthetic fibers (“wool coils”) inserted into the varices prior to cyanoacrylate injection would function as a “barrier” for cyanoacrylate to outflow into the larger veins causing embolic events. In their procedure, after positioning and endosonographic visualization of gastric varices from the esophagus (through the diaphragmatic crus muscle), a 19-gauge FNA needle was inserted through the esophageal wall and diaphragm muscle directly to the gastric varix, following coil delivery and 1 mL of cyanoacrylate with immediate repetition of the procedure as needed until varix obliteration (Figure 1). The treatment was successful in 100% of the patients without complications during the mean 193 days followup, demonstrating the feasibility, efficacy, and safety of this novel method [32]. Weilert et al. in their case record demonstrated successful EUS-guided treatment of rectal varix. A large rectal varix was visualized by endosonography, and two coils following 1 mL of cyanoacrylate were delivered through a 19-gauge needle under direct guidance of EUS, achieving varix obliteration confirmed by Doppler. No bleeding occurred during or after the procedure, leaving the varix obliterated on EUS and Doppler four weeks later, and without recurrent bleeding during one-year followup [35].

EUS-guided management of the upper GI varices and variceal bleeding shares the same theoretical benefits with the EUS-guided management of nonvariceal upper GI bleeding; that is, the visualization of the “culprit” lesions—varices, perforating and collateral veins—allows thorough planning and precise delivery of treatment, as has been demonstrated through the procedures described in the cited papers. Again, in comparison to effective standard treatment modalities, EUS-guided management can offer additional benefit for the patients unsuccessfully treated and those with recurrent bleeding. The quantity of the publications in this area of EUS-guided vascular procedures indicates rapid evolvement, and probably more high quality studies might be expected for these techniques. The studies undertaken up to now underpin the postulated theoretical benefits of EUS-guided therapy over standard techniques, which is most robustly demonstrated in the elective treatment of patients with esophageal collateral veins. 

### 3.3. EUS-Guided Management of Pseudoaneurysms

Visceral pseudoaneurysms are quite rare and serious complications of pancreatitis or abdominal surgery, with high mortality rates when ruptured. Management of visceral pseudoaneurysms includes interventional radiology procedures and surgery, with considerable morbidity and mortality [36, 37]. Percutaneous endovascular procedure is the first-line treatment of these conditions. However, it is often quite difficult to perform, and some aneurysms are practically “unreachable.” Considering the great visualization, and relative proximity of the pseudoaneurysms to the GI tract allowing an attractive access route, EUS-guided procedures offer an alternative to the traditional management options.

Gonzalez et al. in their case record described the patient with chronic pancreatitis having a pseudocyst scheduled for EUS drainage. During the drainage attempt, an intracystic pseudoaneurysm of the splenic artery was injured and massive intracystic hemorrhage occurred. Since this was a potentially life-threatening condition, immediate puncture and cyanoacrylate injection under EUS guidance was performed, embolizing the distal arm of the splenic artery, therefore stopping the bleeding [38]. The patient was followed up for more than a year with no complications recorded. Roberts et al. in their brief report demonstrated successful EUS-guided treatment of visceral pseudoaneurysm by injecting a mixture of Histoacryl glue and lipiodol directly into the lesion [39]. Levy et al. described a patient with a big pseudoaneurysm of the superior mesenteric artery (SMA) that was previously unsuccessfully treated with coiling and injection therapy during angiography. After visualization, a 2 mm wide branch of the SMA communicating with the pseudoaneurysm was injected with 7 mL of 99% alcohol, through a 22-gauge FNA needle, and cessation of blood flow was confirmed by Doppler. There were no complications, and no rebleeding occurred during 16-month followup [16]. 

Roach et al. appear to be the first to describe the use of thrombin for the EUS-guided management of visceral pseudoaneurysm. A 32-year-old man with a superior mesenteric artery (SMA) pseudoaneurysm after pancreatitis and recurrent bleeding episodes was considered for selective embolization during angiography and percutaneous US/CT-guided injection therapy, but these were rejected due to unsuccessful selective catheterization and anatomic positioning. Under the EUS guidance the pseudoaneurysm was punctured with a 22-gauge needle, and 500 IU of thrombin was injected immediately obliterating the sac of pseudoaneurysm leaving the SMA patent, which was confirmed by Doppler. At computed tomography 12 weeks later partial recanalization was verified, but no further treatment was undertaken, and spontaneous rethrombosis of the pseudoaneurysm was detected by computed tomography 28 and 42 weeks after the treatment [40]. Chaves et al. reported a case of a 29-year-old man with a big pseudoaneurysm of the splenic artery at the level of the pancreatic body. The sac of pseudoaneurysm was punctured with a 22-gauge needle under EUS guidance, and 500 IU of thrombin was injected. Obliteration occurred instantly. A week later CT detected a small local splenic infarction, probably due to distal embolization. Four months later CT angiography and EUS confirmed the persistence of the occlusion. The authors hypothesized that the presence of a vascular stalk forming communication between the artery and pseudoaneurysm in this patient was facilitating, increasing the probability of successful obliteration, and reducing the possibility of distal embolization [41]. Robinson et al. also reported the successful treatment of a splenic artery pseudoaneurysm with a 2 mm stalk. Thrombin (500 IU) was injected via a 22-gauge needle into the sac of pseudoaneurysm under the EUS guidance, and occlusion occurred, which was confirmed on an immediate CT angiography and repeatedly one and six weeks later [42]. In their case record Lameris et al. reported the successful EUS-guided injection of 7 mL of thrombin-collagen compound into a splenic artery pseudoaneurysm through a 22-gauge needle, confirming complete pseudoaneurysm obliteration by Doppler, leaving the pseudoaneurysm obliterated at CT angiographies six weeks and ten months after [43].

Publications of rather anecdotal cases of EUS-guided treatment of visceral pseudoaneurysms demonstrated the feasibility and technical ease of performing such procedures and, together with the theoretical benefits over traditional methods, are promising. However, theoretical disadvantages such as bleeding, introduction of infections, unwanted thrombotic events, and efficacy and safety issues, in comparison to standard treatment modalities, should be further robustly evaluated in order to potentially set these methods as the standard.

### 3.4. EUS-Guided Embolization of Portal Venous System

There is some evidence (studies on animal models) that EUS-guided portal vein pressure measurement is feasible, suggesting the EUS-guided transhepatic puncture of portal vein to be safer [44–47]. Additionally, in a study on animal model, the EUS-guided puncture of all major abdominal vessels (arteries and veins) appeared to be feasible and safe [48, 49]. Matthes et al. in their animal model study showed that the EUS-guided embolization of portal vein with ethylene vinyl alcohol is feasible [50], and the same authors in another animal model study demonstrated the feasibility of embolization of splenic vein with ethylene vinyl alcohol (published as abstract). Selective embolization of portal veins can be useful to achieve selective hepatic atrophy before major hepatic surgery [49, 50]. The described EUS-guided procedures offer theoretical therapeutic benefits, but the feasibility and safety issues of such procedures have not been evaluated in humans as of now.

### 3.5. EUS-Guided Creation of Portosystemic Shunt

Portal hypertension of any etiology is associated with substantial complications. Lowering portal vein pressure, thus reducing complication rates, can be achieved with drugs, transjugular intrahepatic portosystemic shunt (TIPSS) placement, or surgical shunts. TIPS is a widely used and effective technique but with a number of possible periprocedural complications, such as pneumothorax formation, cardiac arrhythmias, and injury to the blood vessels in the liver resulting in hemorrhage. The proximity of the portal and hepatic veins to the scope of the EUS offers a potentially more favorable route for shunt formation under EUS guidance, avoiding some of the complications such as pneumothorax and cardiac arrhythmias. However, some potential disadvantages of this approach have to be acknowledged, such as introduction of infection and potentially higher bleeding complications, in comparison to TIPSS. 

**Figure 2:**
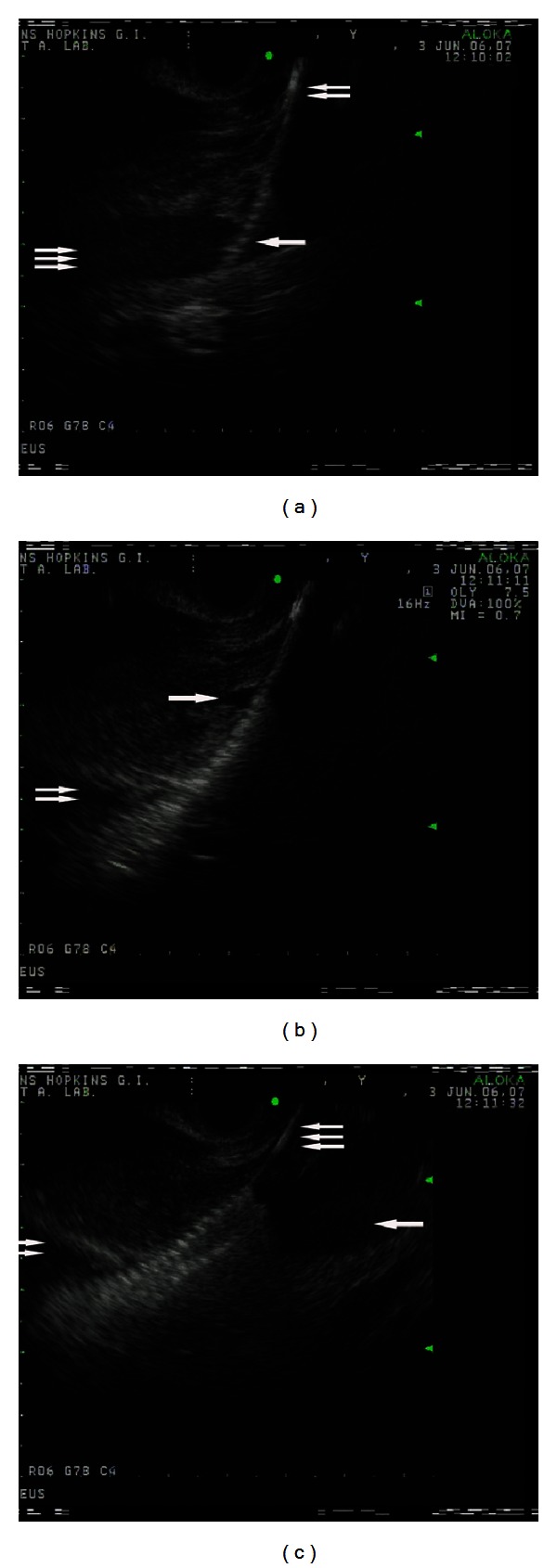
Endosonographic views during the stent deployment. (a) The stent (one arrow) delivery over the guidewire (two arrows) into the hepatic vein (three arrows). (b) Deployment of the stent; the proximal end of the stent inside the hepatic vein (one arrow) and the distal end of the stent inside the portal vein (two arrows). (c) The stent fully deployed with its proximal end inside the hepatic vein (one arrow) and the distal end inside the portal vein (two arrows); the guidewire (three arrows) (images curtsey of [51]).

Recently Buscaglia et al. in their animal model study demonstrated the feasibility of EUS guided creation of an intrahepatic portosystemic shunt (IPSS). After positioning in the plane where both portal and hepatic veins were endosonographically visualized, transhepatic puncture of selected hepatic vein was performed firstly and then advanced to intrahepatic branch of the portal vein with a 19-gauge needle under EUS-guidance aided by fluoroscopy. Hereafter, the stylet was withdrawn following guidewire insertion in the portal vein through the needle, and the needle was withdrawn out from endoscope. After measuring the distance between selected veins, an appropriate uncovered biliary metal stent was inserted over the guidewire, bridging the selected hepatic and portal veins, therefore forming a portosystemic shunt (Figure 2). The authors stated that the procedure was feasible, easy to perform, and effective, without major complications [51]. Further evaluation in humans is needed to evaluate the feasibility, efficacy, and safety, as well as comparison of this theoretically promising alternative to the conventional TIPSS. 

## 4. Conclusion

The paucity of the studies undertaken to date and their quality cannot give answers to the questions posed by clinicians and healthcare policy makers. However, papers published to date can give directions for future research that needs, in a more robust scientific manner, to address technical feasibility, efficacy, and safety of the procedures, as well as cost benefits in this rather young therapeutic arena.

Standard treatment modalities for the management of variceal and nonvariceal GI bleeding as well as for management of visceral pseudoaneurysms and TIPS creation are relatively effective and safe, and as such, are being used worldwide. However, unsuccessful treatment in a percentage of patients and recurrent bleeding episodes, together with theoretical benefits of EUS-guided interventions over standard treatment, as well as feasibility and technical ease demonstrated in the papers summarized in this review, calls for future research.

Further development of EUS-guided access to the vessels could potentially replace the interventional radiology vascular interventions in the abdomen and provide more efficient and precise, as well as safer local application of drugs (chemotherapeutics, fibrinolytics, etc.), vascular embolization (sclerotherapy, coiling, etc.), endoprostheses placement, and creation of portosystemic shunts [48, 49]. 

We all witnessed the “transformation” of ERCP from a purely diagnostic procedure at its beginnings to a mainly therapeutic procedure nowadays [52, 53]. Considering the continuous evolvement of various noninvasive diagnostic modalities and the technical development of EUS, can we expect the same for endoscopic ultrasound in the future?
